# Urine neutrophil gelatinase-associated lipocalin to predict renal response after induction therapy in active lupus nephritis

**DOI:** 10.1186/s12882-017-0678-3

**Published:** 2017-08-04

**Authors:** Bancha Satirapoj, Chagriya Kitiyakara, Asada Leelahavanichkul, Yingyos Avihingsanon, Ouppatham Supasyndh

**Affiliations:** 1 Division of Nephrology, Department of Medicine, Phramongkutklao Hospital and College of Medicine, 315 Rachavitee Road, Phyathai, Bangkok, 10400 Thailand; 2 Division of Nephrology, Faculty of Medicine, Ramathibodi Hospital, Mahidol University, Bangkok, Thailand; 3 Division of Nephrology, Department of Medicine, Faculty of Medicine, Chulalongkorn University, Bangkok, Thailand

**Keywords:** Neutrophil gelatinase-associated lipocalin (NGAL), systemic lupus erythematosus (SLE), lupus nephritis (LN), ROC curve

## Abstract

**Background:**

Tubulointerstitial injury is important to predict the progression of lupus nephritis (LN). Urine neutrophil gelatinase-associated lipocalin (NGAL) has been reported to detect worsening LN disease activity. Thus, urine NGAL may predict renal outcomes among lupus patients.

**Methods:**

We conducted a prospective multi-center study among active LN patients with biopsy-proven. All patients provided urine samples for NGAL measurement by ELISA collected from all patients at baseline and at 6-month follow-up after induction therapy.

**Results:**

In all, 68 active LN patients were enrolled (mean age 31.7 ± 10.0 years, median UPCR 4.8 g/g creatinine level with interquartile range (IQR) 2.5 to 6.9 and mean estimated glomerular filtration rate (GFR) 89.6 ± 33.7 mL/min/1.73 m^2^). At baseline measurement, median urinary NGAL in complete response, partial response and nonresponse groups was 10.86 (IQR; 6.16, 22.4), 19.91 (IQR; 9.05, 41.91) and 65.5 (IQR; 18.3, 103) ng/mL, respectively (*p* = 0.006). Urinary NGAL (ng/mL) correlated positively with proteinuria and blood pressure, and correlated negatively with serum complement C3 level and estimated GFR. Based on ROC analysis, urinary NGAL (AUC; 0.724, 95%CI 0.491–0.957) outperformed conventional biomarkers (serum creatinine, urine protein, and GFR) in differentiating complete and partial response groups from the nonresponse group. The urine NGAL cut-off value in the ROC curve, 28.08 ng/mL, discriminated nonresponse with 72.7% sensitivity and 68.4% specificity.

**Conclusion:**

Urine NGAL at baseline performed better than conventional markers in predicting a clinical response to treatment of active LN except serum complement C3 level. It may have the potential to predict poor response after induction therapy.

## Background

Renal involvement among Asian patients with systemic lupus erythematosus (SLE) in the form of active nephritis is associated with a significant burden of morbidity and mortality [1]. Early detection and prompt treatment with immunosuppressive agents can dramatically change the course of renal disease and improve long-term survival [2, 3]. Current conventional biomarkers have not been very successful in predicting renal histology and patient outcomes. Noninvasive methods should be investigated to assess the renal response with standard treatment among these patients.

Tubulointerstitial damage is important to predict the progression of glomerular diseases and lupus nephritis (LN) [4]. Neutrophil gelatinase-associated lipocalin (NGAL) is a 25-kd protein secreted by leukocytes and tubular epithelial cells under differing conditions with stress and inflammation [5]. Initially, NGAL has been reported in tubular cells and urine in an experimental model of ischemic and nephrotoxic renal injury [6]. Several clinical studies have also demonstrated that concentrations in urine of NGAL represented very sensitive and highly predictive biomarkers for progressive tubular and glomerular injury [7–10]. Moreover, in a study of SLE patients, an increase in urinary levels of NGAL correlated with renal disease activity [11]. Urinary NGAL biomarkers are much easier obtained and might represent a sensitive measurement of kidney injury and local inflammatory activity among LN patients. For these reasons, this study was designed to measure urine NGAL among LN patients and we hypothesized that the levels of urinary NGAL would significantly correlate with the severity of renal disease activity and investigated its predictive performance in renal response after induction therapy.

## Methods

### Population

All SLE patients with biopsy-proven LN class III, IV or V were recruited in the study. Patients fulfilled at least four of the American College of Rheumatology 1982 revised criteria to diagnose SLE. We excluded all patients with drug induced lupus, active malignancies, overlapping syndromes, urinary tract infection, active systemic infection and history of renal transplantation from this study. All patients received standard induction therapy with IV cyclophosphamide or mycophenolate. After 6 months of induction therapy, patients were divided in three groups based on renal response to treatment. These groups of patients with complete response, partial response and nonresponse were defined by return of serum creatinine to previous baseline plus a decline in urinary protein to urinary creatinine ratio (UPCR) to <500 mg/g, stabilization or improved serum creatinine level plus a ≥ 50% decrease in UPCR and sustained 25% increase in serum creatinine level or a < 50% decrease in UPCR, respectively. Informed consent was obtained from all patients who participated in this study.

### Clinical and laboratory measurement

Relevant demographic data of all patients were obtained, as was information on medication regimens. All patients underwent routine laboratory assessments at their outpatient clinic visit. Blood samples were obtained to determine complete blood cell count, serum creatinine level, serum chemistry and serum complement (C3), antinuclear antibodies (ANA) and anti-dsDNA antibody. Estimated glomerular filtration rate (GFR) was assessed using the Chronic Kidney Disease Epidemiology Collaboration (CKD-EPI) equation [12]. A complete urinalysis was performed and the UPCR was obtained.

Disease activity was assessed using the SLE Disease Activity Index (SLEDAI) that “weighted” the index of disease activity in nine organ systems [13]. Renal involvement was assessed with a renal SLEDAI score of 4, corresponding to the presence of any one of the following concerning urine analysis: hematuria, proteinuria, pyuria or urinary red cell casts. The renal histologic features were evaluated by a renal pathologist. Activity index scores were calculated from the summing of individual scores. The range of activity index score was 0 to 24 with higher scores representing higher activity [14]. Chronicity index scores were calculated from the summation of individual scores. The range of chronicity index score was 0 to 12 with higher scores representing higher chronicity [14]. A percentage of each parameter of activity index and chronicity index was calculated by standard method.

### NGAL enzyme-linked immunosorbent assay

Urine samples were collected at baseline and after complete induction therapy. Samples were centrifuged at 4000 rpm for 10 min to remove particular impurities, then stored frozen at −80^∘^C until assayed. Urinary NGAL level was measured by a commercially available sandwich enzyme-linked immunosorbent assay (ELISA) kit from R&D Systems Inc., USA with coefficients of variation for variation less than 10%, for intra-assay and inter-assay variation. The enzymatic reactions were quantified in an automatic microplate photometer. All measurements were made in triplicate and blinded manner. Urinary NGAL excretions were reported as the amount of urinary NGAL in nanograms per milliliter (ng/mL) and urinary NGAL in nanograms per milligram (ng/mg) of urine creatinine to correct for differences in NGAL due to urine dilution.

### Statistical analysis

Statistical analysis was performed using SPSS statistical software, version 16.0 (SPSS, Chicago, IL). Data were given as mean ± SD or median with interquartile range (IQR). Comparisons of categorical variables were conducted using Chi-square testing. For nonparametric data, the differences between groups were analyzed by the Mann–Whitney U and Kruskal-Wallis tests. Spearman correlation coefficients was used as appropriate to test correlations between urine NGAL and other variables. Receiver operating characteristic (ROC) analysis was used to calculate the area under the curve (AUC) with associated 95% confidence interval (CI) for urine NGAL and the usual standard biomarkers used to predict renal response and to find the best cut-off values to identify the renal response after induction therapy. *P* values less than 0.05 were considered significant.

## Results

### Patient characteristics

A total of 68 patients were included in this study, 17 with complete response, 40 with partial response and the other 11 with nonresponse after induction therapy. Most patients were female (97.1%) with a mean age of 31.7 ± 10.0 years. Mean body mass index was within normal range. About 20% of patients had systemic symptoms from SLE. Most of patients were classified as LN class IV defined as proliferative lesion involving 50% or more of glomeruli. Coclassification with class V was found among 14 patients (20.6%). Median score of activity index and chronicity index was 8 (IQR 6–11.5) and 2 (IQR 0–3), respectively. Characteristics of the patients are summarized in Table 1.

**Table 1:** Clinical characteristics

Variables	N (%) or mean ± SD	Median with IQR
Female (N, %)	66 (97.1%)	
Age (years)	31.7 ± 10.0	32 (23, 38)
Body weight (kg)	54.7 ± 14.9	50.9 (45.9, 62)
Body mass index	20.1 ± 3.9	
Duration of SLE (years)	6.3 ± 6.3	4 (2, 8.5)
Systolic blood pressure (mmHg)	126.7 ± 24.3	128 (119, 138.5)
Diastolic blood pressure (mmHg)	79.4 ± 19.2	78.5 (70, 92)
Systemic organ involvements (N, %)		
Arthritis	14 (20.6%)	
Cutaneous lupus	14 (20.6%)	
Hematologic involvement	8 (11.8%)	
Serositis	4 (5.9%)	
Neurological involvement	1 (1.5%)	
Baseline SLEDAI score	10.8 ± 3.3	10 (8, 12)
Baseline Renal SLEDAI score	7.0 ± 2.9	8 (4, 8)
ISN/RPS class (N, %)		
Class III	5 (7.4%)	
Class IV	49 (72.1%)	
Class III + V	4 (5.9%)	
Class IV + V	10 (14.7%)	
Renal activity index	8.5 ± 2.9	8 (6, 11.5)
Renal chronicity index	1.9 ± 1.7	2 (0, 3)
Immunosuppressive agents (N, %)		
Induction with high cyclophosphamide	37 (62.7%)	
Induction with mycophenolate	22 (37.3%)	
Mean dose of mycophenolate (mg/day)	29.6 ± 17.1	30 (20, 35)
Prednisolone	67 (98.5%)	
Hydroxychloroquine	32 (47.1%)	
Anti-hypertensive and lipid lowering agents (N, %)		
RAAS blockers	51 (75%)	
Calcium channel blockers	10 (14.7%)	
Diuretics	28 (41.2%)	
Statins	43 (63.2%)	
Laboratory data		
Positive ANA (%)	67 (98.5%)	
Positive anti-dsDNA (%)	57 (83.8%)	
Low complement component 3 (C3) (%)	31 (60.8%)	
Serum complement component C3 levels (g/L)	1.2 ± 1.8	0.7 (0.4, 1.1)
Hematocrit (%)	26.5 ± 11.2	30 (13.7, 34.6)
Serum albumin (g/dL)	2.7 ± 0.9	3 (2, 3.2)
Serum creatinine (mg/dL)	0.9 ± 0.5	0.8 (0.7, 1.1)
Estimated GFR (mL/min/1.73 m^2^)	89.6 ± 33.7	89.4 (63.7, 116.8)
UPCR (g/g creatinine)	5.9 ± 4.8	4.8 (2.5, 6.9)

*Abbreviations ANA* antinuclear antibody, *Anti-dsDNA* anti-double-stranded DNA antibody, *ISN/RPS* International Society of Nephrology/Renal Pathology Society, *GFR* glomerular filtration rate, *RAAS* renin angiotensin aldosterone system, *SLEDAI* systemic lupus erythematosus disease activity index, *UPCR* urine protein creatinine ratio

Values for categorical variables are given as number (percentage); values for continuous variables, as mean ± standard deviation or median [interquartile range]

### Conventional biomarkers related to renal response

Laboratory biomarker results in the three groups of patients are shown in Table 2. As expected, active LN patients with complete renal response had significantly lower baseline levels of urine protein and renal chronic index, and had significantly higher levels of C3 complements. There was no difference in estimated GFR between the three groups (*P* = 0.467). No significant differences were found regarding age, sex, duration of disease, renal SLEDAI and renal activity pathology score between the three groups of patients. Moreover, there were no significant differences in the baseline conventional biomarkers including urine NGAL between cyclophosphamide and mycophenolate treatment.

**Table 2:** Baseline conventional renal biomarkers with renal response after induction therapy

	Complete response	Partial response	Non-response	*P* value
	(*n* = 17)	(*n* = 40)	(*n* = 11)	
Renal activity index	7 (5, 11)	9 (6, 12)	8 (6, 10)	0.760
Renal chronicity index	0 (0, 3)^a,b^	2 (0, 3)^c^	3 (3, 4)^a,b,c^	0.008
SLEDAI score	12 (10, 14)^a^	10 (8, 12)^a^	12 (8, 16)	0.018
Renal SLEDAI score	8 (4, 8)	4 (4, 8)	8 (8, 12)	0.077
Positive Anti-dsDNA (%)	17 (100%)	39 (97.5%)	11 (100%)	0.705
Serum complement C3 levels (g/L)	0.4 (0.3, 0.6)^a,b^	0.8 (0.5, 1)^a,c^	1.3 (0.9, 1.4) ^a,b,c^	0.001
Hematocrit (%)	29 (13.1, 33.6)	30.3 (11.9, 35.8)	32.9 (14.9, 36)	0.568
Serum albumin (g/dL)	2.9 (2.3, 3.3)	3 (2, 3)	3 (3, 3.2)	0.573
Serum creatinine (mg/dL)	0.8 (0.6, 0.9)	0.9 (0.7, 1.2)	0.7 (0.7, 1.2)	0.553
Estimated GFR (mL/min/1.73 m^2^)	98.5 (85.3, 118.5)	84.2 (59.2, 111.7)	96.2 (53.7, 120.2)	0.467
UPCR (g/g creatinine)	3.1 (2, 4.5)^a^	5.5 (3.9, 8.6)^a^	4.6 (2.1, 6.9)	0.011

*Abbreviations Anti-dsDNA* anti-double-stranded DNA antibody, *GFR* glomerular filtration rate, *RAAS*; renin angiotensin aldosterone system, *SLEDAI* systemic lupus erythematosus disease activity index, *UPCR* urine protein creatinine ratio

Values for categorical variables are given as median [interquartile range]

*P*-value corresponds to ANOVA test, Kruskal-Wallis test (comparison 3 groups) and Mann–Whitney U (comparison 2 groups), ^a^complete response vs. partial response, ^b^complete response vs. nonresponse and ^c^partial response vs. nonresponse

Define term of complete response is return of serum creatinine to previous baseline, plus a decline in the UPCR to <500 mg/g

Define term of partial response is stabilization (±25%), or improvement of SCr, but not to normal, plus a ≥ 50% decrease in UPCR. If there was nephrotic-range proteinuria, improvement requires a ≥ 50% reduction in UPCR, and a UPCR <3000 mg/g

Define term of non-response is a sustained 25% increase in serum creatinine or a < 50% decrease in UPCR

### Baseline urine NGAL with renal activity and renal response

**Fig. 1:**
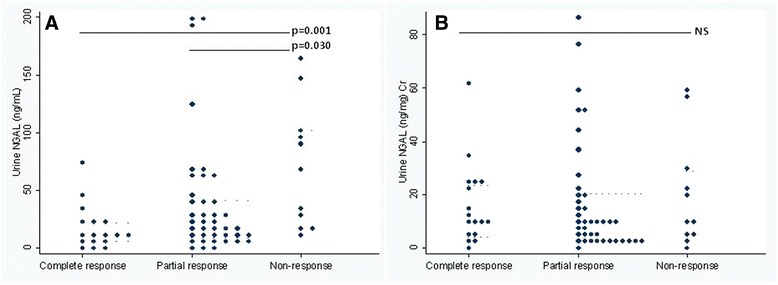
Baseline urinary NGAL among LN patients with renal response after induction therapy **a** Baseline urinary NGAL (ng/mL) among LN patients with renal response after induction therapy and **b** Baseline urinary NGAL to creatinine ratio (ng/mg Cr) among LN patients with renal response after induction therapy

Among active LN patients, baseline urinary NGAL levels were significantly lower among those with complete response (median 10.86 ng/mL, IQR 6.16 to 22.4) than among those with partial response (median 19.91 ng/mL, IQR 9.05 to 41.91), and nonresponse (median 65.5 ng/mL, IQR 18.3 to 103) (*P* = 0.006) (Fig. 1a). However, no significant differences were observed in urine NGAL to urine creatinine ratio among the three groups of patients (Fig. 1b).

To determine whether urinary NGAL was associated with renal activity, we performed a correlation between various baseline renal parameters and level of urine NGAL. Level of urine NGAL (ng/mL) correlated with rising of UPCR (*r* = 0.280, *p* = 0.021), higher systolic blood pressure (*r* = 0.360, *p* = 0.003), higher diastolic blood pressure (*r* = 0.359, *p* = 0.003), lower estimated GFR (*r* = −0.262, *p* = 0.031), and low complement C3 (*r* = −0.389, *p* = 0.005). No correlation was observed between age, positive anti-DNA antibody, SLEDAI score, serum creatinine, and level of urine NGAL (ng/mL). However, level of urine NGAL (ng/mg creatinine) correlated with rising of serum creatinine (*r* = 0.344, *p* = 0.004) and lower estimated GFR (*r* = −0.270, *p* = 0.026) (Table 3).

**Table 3:** Correlation of urine NGAL with other parameters at baseline in active lupus nephritis

	Urine NGAL (ng/mL)		Urine NGAL (ng/mgCr)	
	Spearman’s rho	*p*-value	Spearman’s rho	*p*-value
Age (years)	0.089	0.473	0.001	0.992
ACEIs or ARBs	−0.018	0.883	0.007	0.955
Diuretics	−0.059	0.635	−0.116	0.344
Systolic blood pressure (mmHg)	0.360	0.003	0.198	0.105
Diastolic blood pressure (mmHg)	0.359	0.003	0.045	0.715
SLEDAI score	−0.079	0.523	0.034	0.780
Renal SLEDAI score	0.069	0.576	0.086	0.485
Positive anti-dsDNA antibody	0.040	0.743	−0.171	0.163
Serum complement C3 levels (g/L)	0.273	0.053	0.038	0.793
Low complement C3	−0.389	0.005	−0.130	0.365
Serum creatinine (mg/dL)	0.231	0.058	0.344	0.004
UPCI (g/g creatinine)	0.280	0.021	−0.014	0.910
Estimated GFR (mL/min/1.73 m^2^)	−0.262	0.031	−0.270	0.026

*Abbreviations ACEI,* angiotensin converting enzyme inhibitor, *Anti-dsDNA* anti-double-stranded DNA antibody, ARBs, angiotensin receptor blockers, *GFR* glomerular filtration rate, *RAAS* renin angiotensin aldosterone system, *SLEDAI* systemic lupus erythematosus disease activity index, *UPCR* urine protein creatinine ratio

### Performance of urine NGAL in predicting renal response

**Fig. 2:**
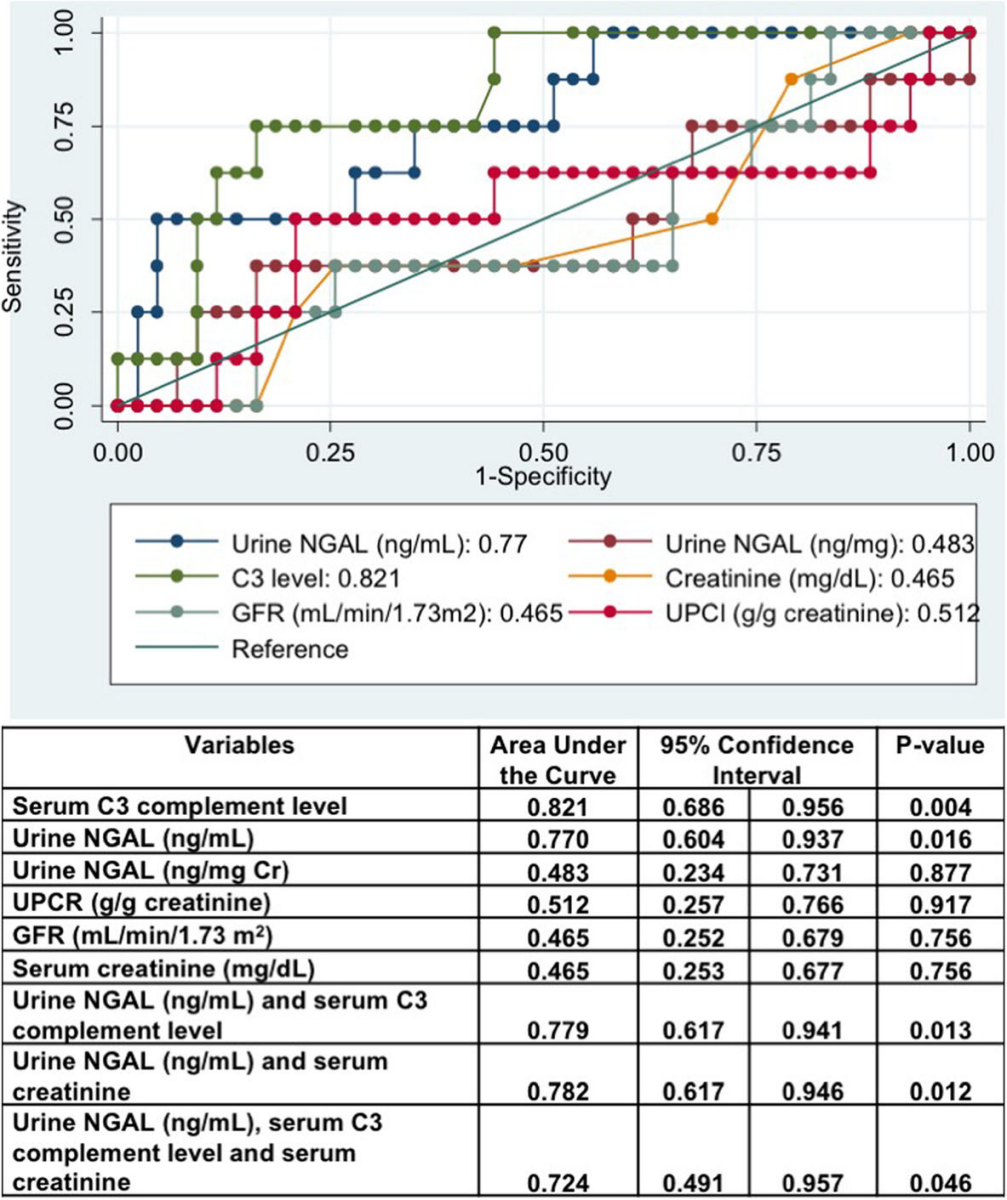
Graph ROC curves showing Area under the Curve (AUC) of urine NGAL to predict renal response after induction therapy. Abbreviations: GFR; Glomerular filtration rate, UPCR; Urine protein creatinine ratio, UNGAL; urine neutrophil gelatinase associated lipocalin

Regarding ROC analysis, urinary NGAL (ng/mL) (AUC; 0.770, 95%CI 0.604–0.937) outperformed conventional biomarkers (serum creatinine, urine protein, and GFR) in differentiating complete and partial response groups from nonresponse group. The AUC for serum C3 complement was 0.821 (95% CI: 0.686–0.956: *P* = 0.004) that was higher than those for urine NGAL. However, the AUCs for urine NGAL (ng/mg creatinine), UPCR, estimated GFR and serum creatinine were 0.483 (*P* = 0.877), 0.512 (*P* = 0.917), 0.465 (*P* = 0.756), and 0.465 (*P* = 0.756), respectively. These were lower than those for urine NGAL (ng/mL) (Fig. 2).

Using the ROC curve data and respective specificity and sensitivity values, optimal thresholds for urine NGAL and complete or partial response after induction therapy were selected. The optimal urine NGAL concentration was 28.08 ng/mL, discriminating nonresponse with 72.7% sensitivity and 68.4% specificity (Table 4).

**Table 4:** Cut-off levels for urine NGAL in predicting renal response after induction therapy

Cut off: Urine NGAL (ng/mL)	Sensitivity	Specificity	PPV	NPV	Accuracy
18.01	81.8%	50.9%	24.3%	93.5%	55.9%
18.45	72.7%	50.9%	22.2%	90.6%	54.4%
18.79	72.7%	52.6%	22.9%	90.9%	55.9%
19.91	72.7%	54.4%	23.5%	91.2%	57.4%
21.40	72.7%	56.1%	24.2%	91.4%	58.8%
22.18	72.7%	57.9%	25.0%	91.7%	60.3%
22.51	72.7%	59.6%	25.8%	91.9%	61.8%
23.01	72.7%	61.4%	26.7%	92.1%	63.2%
23.45	72.7%	63.2%	27.6%	92.3%	64.7%
24.98	72.7%	64.9%	28.6%	92.5%	66.2%
27.24	72.7%	66.7%	29.6%	92.7%	67.6%
28.08	72.7%	68.4%	30.8%	92.9%	69.1%
28.32	63.6%	68.4%	28.0%	90.7%	67.6%
30.16	63.6%	71.9%	30.4%	91.1%	70.6%
34.12	63.6%	73.7%	31.8%	91.3%	72.1%
36.79	54.5%	73.7%	28.6%	89.4%	70.6%
38.92	54.5%	77.2%	31.6%	89.8%	73.5%
41.91	54.5%	78.9%	33.3%	90.0%	75.0%
44.24	54.5%	80.7%	35.3%	90.2%	76.5%
54.21	54.5%	82.5%	37.5%	90.4%	77.9%
63.73	54.5%	86.0%	42.9%	90.7%	80.9%
64.90	54.5%	87.7%	46.2%	90.9%	82.4%
65.45	54.5%	89.5%	50.0%	91.1%	83.8%
66.64	45.5%	89.5%	45.5%	89.5%	82.4%
70.72	45.5%	91.2%	50.0%	89.7%	83.8%
82.96	45.5%	93.0%	55.6%	89.8%	85.3%

## Discussion

Renal involvement that is the main determinant of poor prognosis of SLE supports the need to identify biomarkers to assess the risk of disease development and to follow-up patients with established disease. In recent years, different studies have underlined the crucial role played by the renal tubule in the genesis of progressive acute and chronic glomerulonephritis and its evolution to end stage renal disease [15]. Importantly, in lupus with renal pathology, renal function correlated better with the degree of tubule-interstitial lesions than that of the glomerular lesion. Novel biomarkers of the processes that induce these tubulointerstitial changes may ultimately prove to be better predictors of disease progression and long-term prognosis than our current markers. [4] Findings from the present study clearly indicate that urinary NGAL levels and serum C3 complement might be novel risk markers of LN response to induction therapy beyond the information provided by serum creatinine and other conventional risk factors.

NGAL is a member of the lipocalin family of proteins which is highly expressed in activated leukocytes and other types of cells including tubular epithelial cells with injury [16]. In the initial study, NGAL enhanced excretion in the urine and predicted the future appearance of acute kidney injury in a variety of acute clinical settings [7, 8]. Recent evidence found that NGAL probably induced antibody mediated nephritis by caspase 3 activated apoptosis and up-regulated inflammatory gene expression [17] and also modulated the levels of autoantibodies in animal models of lupus [18]. For all these reasons, NGAL may become one of the most promising next-generation biomarkers in immune mediated nephritis including LN patients. At present, several studies have reported that high levels of urinary NGAL was detected among SLE individuals in the presence of nephritis and urine NGAL may be a predictor of LN disease activity and flares and predicts worsening of LN disease activity [19–21]. In our study, urine NGAL from LN patients related to conventional biomarkers of LN disease activity including urine protein and serum C3 complement. Our finding also showed that active LN with nonresponse to induction therapy had the highest baseline urine NGAL levels, thus providing further evidence for urine NGAL as a predictor for treatment outcome.

Initial data suggested that increased urine NGAL in chronic glomerulonephritis was due to massive protein loss that may saturate the megalin cubilin transporters on the renal tubular cells, leading to reduced NGAL reabsorption [16]. In addition, NGAL mRNA and protein was rapidly induced in the proximal and distal renal tubules in the experimental model of progressive kidney injury [22]. One study among SLE patients also confirmed a significant positive correlation between the urine NGAL level and 24-h urinary protein [23]. Our results were consistent with previous studies [10, 24], revealing a highly significant positive correlation between urine protein and urine NGAL level among all LN patients.

Our study showed no associations between urine NGAL levels with renal function and renal SLEDAI scores for evaluating renal lupus activity. Our findings contrast those reported by other authors suggesting that high urine NGAL reflected the renal components of the SLEDAI score and worsening renal function [25–27]. This discrepancy can be explained by the fact that these authors had compared urine NGAL levels among SLE patients with and without nephritis. However, all patients in our study had biopsy-proven LN without severe renal impairment and limited variation of serum creatinine and renal SLEDAI scores among complete, partial and nonresponse groups.

In our study, the AUCs for serum C3 complement (0.802) and urine NGAL (ng/mL) (0.769) were greater than those for other parameters (proteinuria, serum creatinine and estimated GFR). The sensitivity and specificity in predicting renal response were 75% and 66.7%, respectively. Previous studies among SLE patients have also reported the strong association of urine NGAL [19–21] and serum C3 complement in active LN [28, 29], but data from the Aspreva Lupus Management Study (ALMS) study indicated that only baseline C4 complement level, and early normalization of complement but not C3 complement independently predicted renal response to therapy at 6 months [30]. Proteinuria and estimated GFR are commonly used in clinical practice to evaluate LN activity and response to treatment. Unexpectedly, no significant difference in baseline of serum creatinine and GFR was observed among SLE with and without renal response and we did not find these parameters in predicting LN response to induction therapy. This finding can be explained by the fact that most patients in our study presented active renal disease among SLE patients with limited variation in levels of proteinuria and renal function. Moreover, previous studies used differing reference standards to define renal response or active renal disease activity, and the incongruous reference standards could have led to variance in the outcomes of the present study. In contrast to our study, previous data demonstrated that elevations in serum creatinine and UPCR may reflect ongoing late phase of renal inflammation and were found to be unreliable in predicting the severity of the renal pathology and treatment responses among patients with biopsy-proven LN [31].

Novel urine biomarkers of kidney injury are being studied, and much interest has been shown in the appropriateness and validity of applying urine creatinine normalization. The assumption is that urine creatinine excretion is constant across and within individuals. In our study, the urine NGAL to creatinine ratio was not reflected in the renal response and performed poorly in predicting renal response, but was not detected with urine NGAL (ng/mL). The findings can be explained by the fact that urine creatinine excretion in acute kidney disease is a dynamic process affected by glomerular iltration and tubular secretion, such as urine creatinine decreases in proportion to the magnitude of the decrease in GFR, hence abruptly increasing normalized biomarker levels [32]. Nevertheless, normalized levels of a urine biomarker such as NGAL reflecting tubular injury can be influenced by dynamic changes in the urine creatinine excretion rate [33]. Therefore, creatinine normalization might be inappropriate for progressive kidney injury, especially among our patients receiving aggressive immunosuppressive treatment in active LN.

Study limitations included a relatively short follow-up period of 6 months and no comparison of main renal outcomes to demonstrate the doubling of serum creatinine and initiating long-term dialysis. The relatively small sample size in our study limited the precision and power to detect associations of moderate strength. Differences in immunosuppressive agents used throughout the study could not be controlled, measurement of blood level of mycophenolate was not done and their influence on our data remains uncertain. Finally, follow-up was based on a serum creatinine level estimated GFR and urine protein for defining renal response, instead of renal biopsy, which may have mildly deviated renal outcomes.

## Conclusion

In conclusion, this study indicates that NGAL in urine is one of biomarkers of the tubulointerstitial changes and perform better than conventional markers in predicting a clinical response to treatment of active LN. It may have the potential to predict renal response after induction therapy among SLE patients. Further investigation should focus on large populations with an analysis of urinary NGAL to find out whether NGAL can be used in clinical practice with higher sensitivity and specificity in predicting long term renal outcomes among SLE patients.

## Abbreviations

ALMS: Aspreva Lupus Management Study
ANA: Antinuclear antibodies
AUC: Area under the curve
CI: Confidence interval
CKD-EPI: Chronic Kidney Disease Epidemiology Collaboration
ELISA: Enzyme-linked immunosorbent assay
GFR: Glomerular filtration rate
IQR: Interquartile range
LN: Lupus nephritis
NGAL: Neutrophil gelatinase-associated lipocalin
ROC: Receiver operating characteristic
SLE: Systemic lupus erythematosus
SLEDAI: SLE Disease Activity Index
UPCR: Urinary protein to urinary creatinine ratio
